# Post-pulmonary metastasectomy prognosis after curative resection for colorectal cancer

**DOI:** 10.18632/oncotarget.16616

**Published:** 2017-03-28

**Authors:** Jee Yeon Kim, In Ja Park, Hyeong Ryul Kim, Dong Kwan Kim, Jong Lyul Lee, Yong Sik Yoon, Chan Wook Kim, Seok-Byung Lim, Jung Bok Lee, Chang Sik Yu, Jin Cheon Kim

**Affiliations:** ^1^ Department of Colon and Rectal Surgery, University of Ulsan College of Medicine and Asan Medical Center, Seoul, Korea; ^2^ Department of Thoracic and Cardiovascular Surgery, University of Ulsan College of Medicine and Asan Medical Center, Seoul, Korea; ^3^ Department of Clinical Epidemiology and Biostatistics, University of Ulsan College of Medicine and Asan Medical Center, Seoul, Korea

**Keywords:** colorectal cancer, pulmonary metastasectomy, disease free survival

## Abstract

**Purpose:**

We aimed to compare disease-free survival after pulmonary metastasectomy to that after hepatic metastasectomy, and to identify prognostic factors after pulmonary metastasectomy.

**Results:**

Between 2005 and 2015, 129 patients underwent resection of isolated metachronous lung metastases from colorectal cancer. Three-year DFS after pulmonary metastasectomy was similar to that after hepatic metastasectomy (50.7% vs. 45.5%, respectively; p=0.58). Rectal cancer (hazard ratio [HR]: 2.04, 95% confidence interval [CI]: 1.09–3.79; p=0.03) and ≥2 metastases (HR: 2.17, 95% CI: 1.28–3.68; p=0.004) were independent adverse risk factors associated with disease-free survival after pulmonary metastasectomy on multivariate analysis. Three-year DFS for colon vs. rectal cancer patients was 72.5% vs. 42.6%, respectively (p=0.04). The number of lung metastases was an independent risk factor for DFS after pulmonary metastasectomy in rectal cancer patients.

**Patients and Methods:**

Patients who underwent lung metastasectomy after curative resection of colorectal cancers were investigated. Disease-free survival (DFS) after pulmonary metastasectomy was compared to that after hepatic metastasectomy, which has a relatively well-known prognosis. Multivariate Cox proportional hazards analysis was performed to identify clinical variables predictive of survival after pulmonary metastasectomy.

**Conclusions:**

Disease-free survival rates after resection of lung vs. liver metastases arising from colorectal cancers are similar. However, lung metastases specifically from rectal cancers produce poorer DFS rates. Primary tumor location must be considered for pulmonary metastasis treatment and follow-up in colorectal cancer patients.

## INTRODUCTION

Metastatic disease develops in 50% of colorectal cancer patients [1]. Resection of liver metastases from colorectal cancers has evolved into a standard treatment option after case series and clinical trials demonstrated that a substantial proportion of patients achieve long-term survival as a result [2]. Five-year survival after hepatic metastasectomy is reported to approach 30–40%; a recent meta-analysis reported a median 5-year survival rate of 38% [3]. Lung is the one of the most common site of metastasis from colorectal cancer, and pulmonary metastasectomy has been rapidly suggested as a potentially curative option in multimodal management of metastatic colorectal cancer [4]. However, it remains unclear why metastasectomy would be beneficial against hematogenous metastatic disease [5]. Case series from leading cancer centers suggest that approximately 30–55% of patients who undergo resection of CRC lung metastases will achieve long-term survival [5, 6]. Yet there is considerably less evidence in favor of resecting colorectal cancer lung metastases when compared to resecting liver metastases although lung metastases is as common as liver metastases [2]. Furthermore, there is no consensus on identifying patients with lung metastases who may most benefit from metastasectomy; current data are derived from short randomized controlled trials [7].

Previous reports on pulmonary metastasectomy for colorectal cancer have proposed several prognostic factors. However, results vary among studies, and clinical utilization of these prognostic factors has not yet been implemented [6].

Hence, the purpose of this study was to compare disease-free survival (DFS) after pulmonary metastasectomy to that after hepatic metastasectomy because oncologic outcome after hepatic meastasectomy from colorectal cancer is relatively well known. We also aimed to identify factors predictive of prognosis after pulmonary metastasectomy.

## RESULTS

### Characteristics of patients

Characteristics of the 129 patients (77 men and 52 women) included in the study are shown in Table 1. The mean age at metastasectomy was 56 years. Rectal cancer comprised of 70.5% of the cases. DFIs ranged from 6 to 66 months; 14 patients (10.9%) had a DFI of less than 1 year. Among the 91 patients with rectal cancer as their primary malignancies, 59.3% received preoperative chemoradiotherapy prior to primary tumor surgery. The vast majority of patients (94.6%) received adjuvant chemotherapy for primary colorectal cancer.

**Table 1:** Patients’ demographics and characteristics of primary colorectal cancer

**Mean age at metastasectomy**	56 [33–76]
**Gender**	
Male	77 (59.7)
Female	52 (40.3)
**Location of primary cancer**	
Colon	38 (29.5)
Rectum	91 (70.5)
**DFI to lung metastasis**	
<12 months	14 (10.9)
≥12 months	115 (89.1)
**Primary stage**	
0 (NRT)	4 (3.1)
I	19 (14.7)
II	44 (34.1)
III	62 (48.1)
**PCRT for primary rectal cancer**	54/91 (59.3)
**Adjuvant chemotherapy for primary cancer**	
None	7 (5.4)
5-FU based	56 (43.4)
Capecitabine	52 (40.3)
Others	11 (8.5)
Not known	3 (2.3)

Data are presented as *n* [range] or *n* (percent).

DFI = disease-free interval; NRT = no residual tumor; PCRT = preoperative chemoradiotherapy; FU = fluorouracil.

### Characteristics and treatment of pulmonary metastases

The characteristics and treatments of the pulmonary metastases in our patients are shown in Table 2. Eighty-six patients (66.7%) presented with lung lesions 1 cm or larger; the largest excised lesion was 6.0 cm.

**Table 2:** Characteristics and treatment of lung metastases

**Location of lung metastasis**	
Unilobar	120 (93.0)
Bilobar	9 (7.0)
**Number of lung metastasis**	
Single	101 (78.3)
Multiple (≥2)	28 (21.7)
**Size of lung metastasis**	
< 1cm	43 (33.3)
≥ 1cm	86 (66.7)
**Type of resection**	
Wedge resection	105 (81.4)
Segmentectomy	11 (8.5)
Lobectomy	9 (7.0)
Combined	4 (3.1)
**CEA level before lung resection**	
Normal	115 (89.1)
Elevated (> 5ng/ml)	14 (10.9)
**Resection margin involvement after lung resection**	1 (0.8)
**Neoadjuvant chemotherapy for lung metastasis**	
Yes	9 (7.0)
No	120 (93.0)
**Adjuvant chemotherapy after lung resection**	
Yes	99 (76.7)
No	30 (23.3)
**Follow up duration after lung resection, months**	46.4 [9–111]

Data are presented as *n* [range] or *n* (percent).

CEA = carcinoembryonic antigen.

During pulmonary metastasectomy, lymphadenectomy was performed in 34 cases (26.4%); 6 patients were found to have thoracic lymph node involvement. After metastasectomy, 99 patients (76.7%) received adjuvant chemotherapy for lung metastases; the most common regimen was capecitabine plus oxaliplatin (XELOX, 37.4%), followed by capecitabine alone (30.3%), 5-fluorouracil and leucovorin plus oxaliplatin (FOLFOX, 18.2%), 5-fluorouracil and leucovorin plus irinotecan (FOLFIRI, 11.1%), and capecitabine plus irinotecan (XELIRI, 2.0%). A single patient received irinotecan alone (0.8%). Neoadjuvant chemotherapy was recommended to patients with multiple lesions or nodal involvement detected on imaging studies. Hence, 9 patients (7.0%) received chemotherapy for lung metastases prior to surgery (3 received 12 cycles of FOLFOX and 1 received 4 cycles). The remaining 5 patients received 3 cycles of XELOX; a single cycle of capecitabine alone; 8 cycles of XELIRI followed by 3 cycles of tegafur-uracil; 21 cycles of irinotecan in combination with tegafur-uracil and cetuximab; and 12 cycles of FOLFIRI followed by a combination of radiation therapy and 8 cycles of FOLFOX due to patient's initial refusal to undergo surgery; respectively.

### Oncologic outcome and risk factors

During a mean follow-up period after metastasectomy of 46.4 months (range, 9–111 months), 65 patients (50%) showed recurrence. The most common recurrence site was the lung (46 patients, 70.8%) followed by the liver (9 patients, 13.8%); distant lymph nodes (7 patients, 10.8%); and other systemic metastases in the brain, bone, and thyroid (8 patients, 12.3%). Three patients (4.7%) showed local recurrence at the site of the primary colorectal cancer.

Among the 46 patients who again developed recurrence in the lung after pulmonary metastasectomy, 26 (56.5%) underwent another lung metastases resection. The remaining 20 patients (43.5%) were either ineligible for surgery due to multiple lesions, location of the metastases, or comorbidities; found to have other distant metastases; or reluctant to undergo surgery. These patients were subjected to systemic chemotherapy (8 patients), radiation therapy to the lung combined with or followed by systemic chemotherapy (6 patients), watchful waiting (3 patients), radiation therapy to the lung alone (2 patients), or Cyberknife treatment (1 patient).

**Figure 1:**
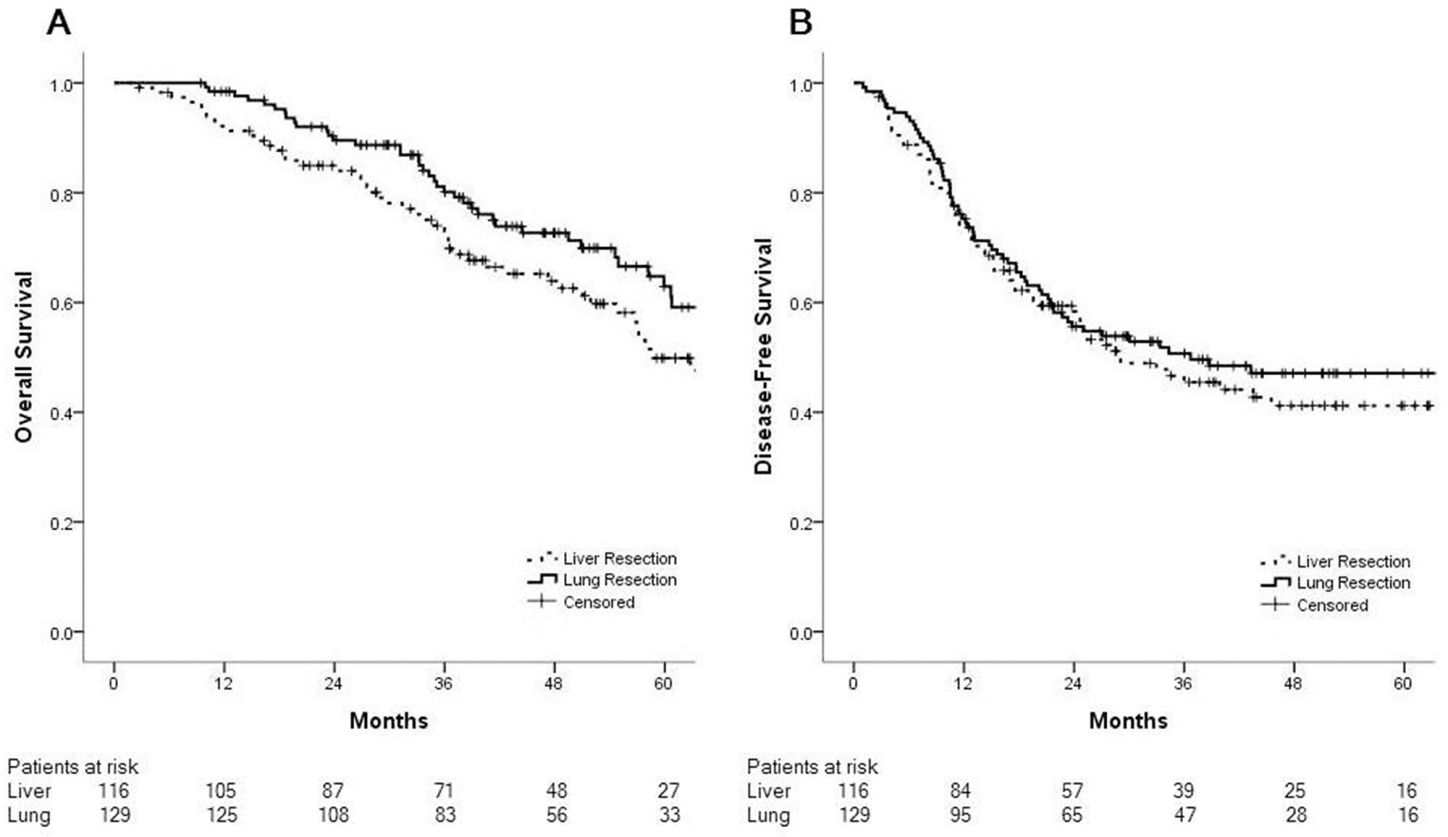
Overall survival and disease-free survival after pulmonary (n=129) and hepatic (n=116) metastasectomies are similar **(A)** Five-year overall survival rates after pulmonary metastasectomy and hepatic metastasectomy were 62.9% and 49.8%, respectively (p=0.13). **(B)** Three-year disease-free survival rates after pulmonary metastasectomy and hepatic metastasectomy were 50.7% and 45.5%, respectively (p=0.58).

The 3-year DFS rate after pulmonary metastasectomy was 50.7%; this rate was similar to that after hepatic metastasectomy (p=0.58). 5-year OS rate after pulmonary metastasectomy and hepatic metastasectomy were 62.9% and 49.8%, respectively (p=0.13) (Figure 1).

Rectal cancer was significantly associated with poor outcome after pulmonary metastasectomy (p=0.02). Other than the location of the primary tumor, the number of lung metastasis lesions (single versus multiple) was the only factor significantly associated with disease-free survival on both univariate and multivariate analyses (p=0.004). Size of lesion (smaller or larger than 1 cm), DFI, and adjuvant chemotherapy after lung metastasectomy had no significant impact on outcome (Table 3).

**Table 3:** Univariate and multivariate analysis of factors associated with disease-free survival after pulmonary metastasectomy

	Univariate			Multivariate		
	HR	(95% CI)	p	HR	(95% CI)	p
**Location of primary tumor**			0.02			0.03
Colon	1			1		
Rectum	2.06	(1.10-3.84)		2.04	(1.09-3.79)	
**No. of lung metastasis**			0.005			0.004
Single	1			1		
Multiple (≥2)	2.14	(1.25-3.64)		2.17	(1.28-3.68)	
**DFI to lung metastasis**			0.12			0.07
≥12 mon	1			1		
<12 mon	1.76	(0.87-3.59)		1.88	(0.96-3.70)	
**Size of lung metastasis**			0.96			
<1cm	1					
≥1cm	0.99	(0.58-1.67)				
**CEA level before lung resection**			0.53			
normal	1					
elevated (> 5ng/ml)	1.30	(0.57-2.93)				
**Adjuvant chemotherapy after lung resection**			0.53			
No	1					
Yes	0.73	(0.41-1.31)				

HR = hazard ratio; CI = confidence interval; DFI = disease-free interval; CEA = carcinoembryonic antigen.

### Outcome of pulmonary metastasectomy in rectal cancer

**Figure 2:**
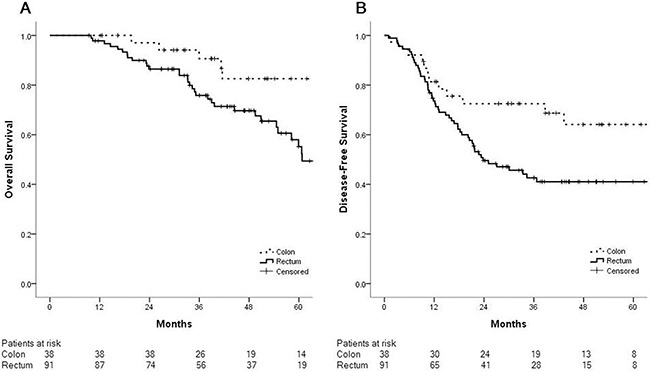
Patients who underwent pulmonary metastasectomy after resection of primary rectal cancer had poorer outcomes than those after resection of primary colon cancer **(A)** The five-year overall survival rate after pulmonary metastasectomy in rectal cancer patients was 55.2% compared to 82.6% in colon cancer patients (p=0.005). **(B)** The three-year disease-free survival rate after pulmonary metastasectomy in rectal cancer patients was 42.6% compared to 72.5% in colon cancer patients (p=0.04).

Our analyses showed that pulmonary metastasectomy of rectal cancer is associated with poorer outcome. We compared clinical characteristics and treatment between the 91 rectal cancer vs. 38 colon cancer patients in our study, and found no difference between the 2 groups (Supplementary Table 1). The 3-year disease-free survival was significantly poorer in patients who received pulmonary metastasectomy due to rectal cancer than in patients due to colon cancer (42.6% vs. 72.5%, respectively; p=0.04). The 5-year OS after pulmonary metastasectomy in rectal cancer patients was 55.2% compared to 82.6% in colon cancer patients (p=0.005) (Figure 2). We separately analyzed factors affecting disease-free survival among the 91 rectal cancer patients (Table 4). Having multiple lung metastases lesions was the only factor to have an independent impact on poor outcome on univariate and multivariate analyses (p=0.005).

**Table 4:** Univariate and multivariate analysis of factors associated with disease-free survival after pulmonary metastasectomy in rectal cancer

	Univariate			Multivariate		
	HR	(95% CI)	p	HR	(95% CI)	p
**No. of lung metastasis**			0.008			0.005
Single	1			1		
Multiple (≥2)	2.30	(1.25-4.24)		2.34	(1.29-4.24)	
**DFI to lung metastasis**			0.24			
≥12 mon	1					
<12 mon	1.63	(0.72-3.65)				
**Size of lung metastasis**			0.55			
<1cm	1					
≥1cm	0.84	(0.47-1.49)				
**CEA level before lung resection**			0.44			
Normal	1					
Elevated (> 5ng/ml)	1.46	(0.56-3.81)				
**Adjuvant chemotherapy after lung resection**			0.80			
No	1					
Yes	0.92	(0.49-1.74)				

HR = hazard ratio; CI = confidence interval; DFI = disease-free interval; CEA = carcinoembryonic antigen.

### Molecular analyses and biologic agents

Majority of the population were tested for microsatellite instability (MSI) and 82.2% of patients were microsatellite-stable (MSS). KRAS wild-type was detected in 31 of 50 patients examined for mutational status of KRAS. Among 41 patients evaluated for BRAF gene status, only a single patient who received pulmonary metastasectomy from rectal cancer had BRAF mutation (Table 5). Combined with adjuvant chemotherapy after pulmonary metastasectomy, two patients received bevacizumab and one patient received cetuximab as biologic agents. Among the 65 patients who again developed once or more recurrence after metastasectomy, 11 patients received bevacizumab, 5 received cetuximab, 2 received panitumumab treatment regardless of their recurrence site.

**Table 5:** Molecular analyses for MSI status and KRAS, BRAF gene mutations

**MSI status**	
MSS	106 (82.2)
MSI	2 (1.5)
Missing	21 (16.3)
**KRAS**	
Wild-type	31 (24.0)
Mutated	19 (14.7)
Missing	79 (61.2)
**BRAF**	
Wild-type	40 (31.0)
Mutated	1 (0.8)
Missing	88 (68.2)

Data are presented as *n* (percent).

MSI = microsatellite instability; MSS = microsatellite-stable.

## DISCUSSION

This study reports the management and outcomes of resected metachronous lung metastases arising from colorectal cancers at a single center during a 9-year period. Fifty percent of patients who underwent resection for colorectal cancer lung metastases showed recurrence, mostly in the lung, with a 3-year DFS rate of 50.7%. The DFS rates after pulmonary and hepatic metastasectomy were similar. Moreover, patients with lung metastases from rectal cancer had significantly poorer 3-year DFS than those with metastases from colon cancer. Multiple lung metastatic lesions were found to be an unfavorable prognostic factor in both the entire study population and among the rectal cancer lung metastases group.

While there is no prospective randomized study on lung metastasectomy in colorectal cancer, our study is the first to present outcomes of isolated metachronous pulmonary and hepatic metastasectomies performed under identical conditions. Previous retrospective studies and systematic reviews compared patients with isolated liver metastases undergoing hepatic resection with untreated patients or those who underwent palliative chemotherapy, and reported prolonged median survival after resection [8]. Hence, liver resection is currently the only treatment modality that potentially achieves long-term survival and offers the possibility of a cure [9]. Patients who achieve R0 resection of liver metastases have 5- and 10-year overall survival rates of approximately 40% and 25%, respectively [10]. It has been postulated that, because hematogenous spreading usually occurs in a stepwise fashion – initially to the liver, with subsequent intrahepatic spread via the portal vein and further spreading to systemic circulation – surgical resection of isolated hepatic metastases from colorectal cancer may be curative [8]. Iida et al. investigated recurrence sites after pulmonary metastasectomy and proposed that metastases to the lungs or other upstream organs (i.e., locoregional sites or the liver) can be regarded as semi-local disease [6]. The possibility that the lung can anatomically or mechanically filter cancer cells has been hypothesized based on experimental animal studies and autopsies [11, 12]. Our study also reported similar DFS rates for hepatic and pulmonary metastasectomy patients, supporting the notion that if pulmonary metastasis is a semi-local recurrence, pulmonary metastasectomy ought to be indicated as long as all lesions are resectable.

In a 30-year population-based study, Mitry et al. reported that rectal cancer had a higher risk of developing lung metastasis than colon cancer [13]. A few series have reported a higher incidence of isolated lung metastasis in patients with rectal cancer compared to patients with colon cancer [13, 14]. A recent study by Cho et al. compared outcomes after pulmonary metastasectomy in 346 colon cancer and 280 rectal cancer patients, and reported poorer 5-year disease-free survival in the latter group (60.1%) than in the former (67.2%) [15]. Our data revealed a larger difference in 3-year DFS after pulmonary metastasectomy between rectal cancer (42.6%) and colon cancer (72.5%) patients. However, others including Cho et al. reported no impact of the primary tumor location on overall survival [15, 16]. While several theories have been proposed to explain these results, the reasons are likely multifactorial. For example, anatomical factors such as vasculature might influence metastatic patterns; since venous return from the rectum transits through the iliac system, rectal cancer can metastasize more easily than colon cancer to the systemic rather than the portal circulation, resulting in a higher incidence of spreading to the lung [17, 18]. On the contrary with the present study, the large randomized prospective study presented recently showed better oncologic outcomes of left colon cancer including rectal cancer and it is contradictory to our results [19, 20]. The patients in their study were different from our cohort. They included colorectal cancer patients with unresectable metastatic colorectal cancer treated with target agent. Therefore, extensiveness of metastatic disease which is hard to be measured numerically would influence on oncologic outcomes although they analyzed data in subset of patients who had similar molecular basis. In addition, the effect of therapeutic agent would also influence on oncologic outcomes. Our cohort was patients with metachronous isolated pulmonary metastasis which could be indicated for surgical resection. However, the limitation of the present study such as the lack of molecular status which has been reported as a significant independent prognostic factor in colorectal cancer would influence on the analysis of data and induce biased results. Molecular characteristics may influence the site of metastasis; some genetic alterations may be associated with metastasis to specific sites, and the site of metastasis may in turn influence the stromal expression profile and its concordance with the primary tumor [19–21]. The prognostic impact of tumor location in cohort of the present study needs to be evaluated further via the other large scale study.

Other than the location of the primary tumor, having multiple lung metastases was the only factor to significantly influence survival after pulmonary metastasectomy on both univariate and multivariate analyses. In a meta-analysis of 25 published series by Gonzalez et al., 13 studies identified multiple pulmonary metastases as a prognostic factor of poor clinical outcome compared to solitary pulmonary metastasis [22]. A recent meta-analysis of the Spanish pulmonary metastasectomy registry (GECMP-CCR) showed a doubling risk of poor outcome due to multiple metastases [23]. We cautiously propose that, as long as R0 resection is feasible, the presence of multiple lung metastatic lesions can be used as a prognostic factor not to select or exclude patients from surgical indications but rather to identify patients more likely to benefit from surgery.

The recurrence rate following initial metastasectomy for patients with lung metastases has been shown to approach 68%; the lung is the most common site of recurrence [21]. Our results are consistent with these findings, with a 50% recurrence rate after lung metastasectomy among which 70.8% recurred in the lung. There is no clear guidance on which patients may benefit from repeat resection; however, if the metastases are operable, it has been reported that select patients may benefit from multiple metastasectomies, with a 5-year overall survival of 25–58% [5, 24]. In our study, the repeat resection rate for pulmonary recurrence was 56.5%.

The majority of patients in our study have received VATS resection. It is debatable whether VATS resection could be appropriate for metastasectomy since it could overlook nodules otherwise palpable during open thoracotomy. It is usually admitted that there is a bad concordance between the number of nodules observed at the CT-scan and the number of resected nodules by thoracotomy (frequently higher) in cases of lung metastases of colorectal cancer, more particularly when there is more than 1 nodule [25]. It is hard to compare oncologic outcome between operative methods because the number of patients who underwent thoracotomy was too small to compare with those with VATS. Among patients who treated with VATS, 46 patients who again developed recurrence in the lung, 29 cases (63.0%) did not show any evidence of re-recurrence during the first 12 months thus concluded as R0 resection of metastases. However, 17 patients had disease-free interval to re-recurrence of less than 12 months and notably, nine patients did not show other lesions on their chest CT scan diagnosing the first lung metastases. Therefore, we thought that VATS could be done with oncologic safety in the selected patients.

Several limitations exist in this study, including its retrospective design within a small population from a single institution and the relatively short length of the follow-up period. Thoracic nodal involvement is an aspect often analyzed in retrospective trials and is observed to be associated with an increased risk of death. However, we did not include nodal status as a parameter in our analyses because of the low rate of nodal dissection during metastasectomy (26.4%). This may be because majority of patients received resection through video-assisted thoracoscopy and did not routinely perform hilar and mediastinal node dissection for metastatic cancers [22]. So far, among molecular markers studied, MSI status and RAS, BRAF mutations represent important predictive factors of response to target agents in metastatic colorectal cancer [26, 27]. KRAS and BRAF status were unavailable in more than half of the cases and thus led to overlooking confounding factors which may have contributed to survival. In addition, because of the heterogeneity of chemotherapy regimen, it is difficult to demonstrate influence of perioperative treatment.

Operative indications should be intrinsically decided by comparing outcomes among patients of the same condition who underwent pulmonary resection vs. those who did not. The results of randomized trials such as the pulmonary metastasectomy in colorectal cancer (PulMiCC) underway in Europe will be a key to determining whether pulmonary metastasectomy improves survival in colorectal cancer [28].

In conclusion, metastasectomy for metachronous lung metastases after resection of primary colorectal cancer shows outcomes similar to those observed after hepatic metastasectomy, and may therefore offer benefits for DFS in selected patients. Impact of location of primary tumor on prognosis in patients received pulmonary metastasectomy from colorectal cancer needs to be evaluated in the further study because of the potential bias caused by retrospective nature and lack of molecular consideration of the present study. Multiple lung metastatic lesions are potential prognostic factors affecting outcome after pulmonary metastasectomy; surveillance and management can be optimized by considering these factors.

## MATERIALS AND METHODS

### Patients and data collection

**Figure 3:**
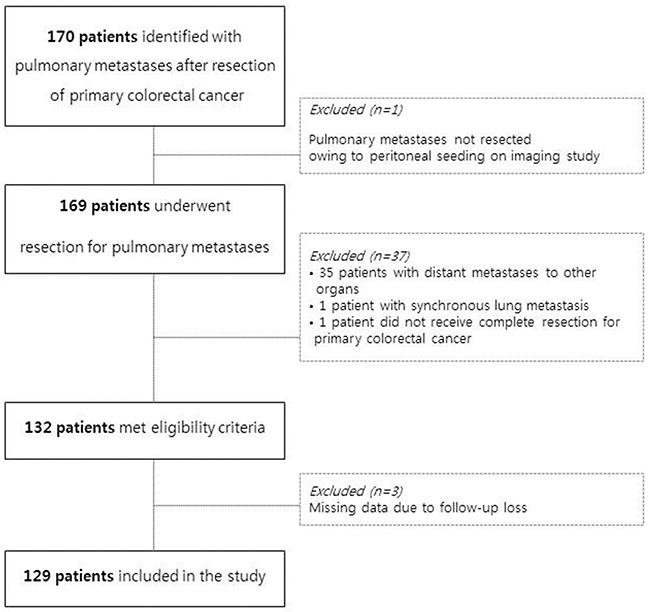
Quorum flowchart of the study design

Between January 2005 and December 2014, 170 patients with pulmonary metastases from primary colorectal cancers were extracted from the Tumor Registry of Asan Medical Center. Among them, 169 patients had received surgical resections for pulmonary metastases. We excluded patients who also had metastases to organs other than the lung, those who received resection for synchronous pulmonary metastases, and those who did not receive curative surgery for the primary colorectal cancer. Ultimately, 129 eligible patients were included in the present study (Figure 3). The medical records of these 129 patients who underwent resection of metachronous pulmonary metastases were reviewed; patients’ demographic information, primary colorectal tumor characteristics, and pulmonary metastases treatment details were collected. For comparison, patients who underwent hepatic metastasectomy were also recruited; 116 patients who received hepatic metastasectomy for metachronous liver metastases after curative resection of primary colorectal cancer with no other systemic metastases during the same time period were included in the analyses.

Follow-up after resection for primary cancer was conducted through outpatient visits and consisted of physical examination, measurement of serum carcinoembryonic antigen (CEA) levels, and chest radiography every 3–6 months. Additionally, abdominopelvic and chest computed tomography (CT) scans with or without colonoscopy were performed every 6–12 months. CT scans were acquired earlier upon suspicious chest radiography findings or elevated serum CEA levels.

### Diagnosis and treatment of pulmonary metastasis

Lung metastases were diagnosed when suspicious lesions were observed to be growing on serial chest CTs with or without confirmation of high uptake on positron emission tomography (PET)-CT. If the lung lesions were unclear, biopsies were performed when feasible. Thirteen biopsies were performed for lung lesions; 2 cases were negative for malignancies, however, serial chest CT with PET-CT results, as well as elevated serum CEA levels, indicated lung metastases in both of these patients.

Metachronous disease was defined as metastases detected 6 months or more after definitive treatment or curative resection of the primary colorectal cancer [8]. Prior to treatment, evaluation of distant metastases to other organs was performed using imaging studies and biopsies, if necessary. For patients with other resectable distant metastases, alternative serial surgical treatment plans were discussed as these patients were excluded from our study.

Nonanatomical wedge resection was the preferred surgical option for the majority of patients; however, lobectomy or segmentectomy was also performed to achieve complete (R0) resection according to the location or multiplicity of the lesions. There were 107 cases (82.9%) that underwent resection through video-assisted thoracoscopy. Nodal status was evaluated at the surgeon's discretion during metastasectomy, and sampling or removal of nodules was usually performed because of increased suspicion following preoperative imaging test results.

A majority of patients received chest radiography or chest CT every 3 months for 1 year after pulmonary resection. Some patients with suspicious lesions in other organs were also monitored with PET-CT every 6 months during the first year to detect local and systemic tumor relapse. Systemic perioperative chemotherapy was determined by a multidisciplinary board of oncologists, surgeons, radiologists, and radiotherapists. Recurrence after surgical treatment of pulmonary metastases was determined based on radiological or histopathological findings, similar to the methods used to diagnose initial pulmonary metastases. Each patient provided informed consent before treatment.

The study was conducted after approval by the local Human Investigations Committee and in accordance with the Declaration of Helsinki; informed consent was waived. This study was approved by the Ethics Review Board of Asan Medical Center.

### Statistical analysis

The clinical characteristics of the study patients were compared using the Pearson's chi-square test, Fisher exact test, or student t test, as applicable. Disease-free interval (DFI) was defined as the interval between the resection of the primary colorectal cancer and date of diagnosis of pulmonary metastasis. DFS was calculated from the date of pulmonary metastasectomy to the date of first recurrence at any site or death. Overall survival (OS) was assessed from the pulmonary metastasectomy to the time of death or last follow-up.

DFS and OS curves were plotted using the Kaplan-Meier method, and differences between groups were assessed with the log-rank test. The following factors were evaluated for their influence on disease recurrence in pulmonary metastasectomy patients: primary tumor location, interval to lung metastases, number of lung metastasis lesions, size of lung metastasis lesions, and adjuvant chemotherapy after resection. A Cox proportional hazards model was used in both univariate and multivariate analyses to identify factors associated with DFS and all factors which p values < 0.2 in univariate analysis were included in multivariate analysis. A probability value of less than 0.05 was considered statistically significant. All statistical analyses were performed using SPSS (Version 21.0, IBM statistics, Armonk, NY).

## SUPPLEMENTARY TABLE





## Abbreviations

CRC: colorectal cancer
CT: computed tomography
DFI: disease-free interval
DFS: disease-free survival
MSI: microsatellite instability
MSS: microsatellite stable
OS: overall survival
HR: hazard ratios
