# Supplementary material for: Post-pulmonary metastasectomy prognosis after curative resection for colorectal cancer
Source: Oncotarget. 2017 Mar 28;8(22):36566–77. doi: 10.18632/oncotarget.16616 (PMC5482677; doi:10.18632/oncotarget.16616)
Supplement: Supplementary file 1 [file oncotarget-08-36566-s001.pdf]

Post-pulmonary metastasectomy prognosis after curative resection for colorectal cancer

SUPPLEMENTARY MATERIALS

SUPPLEMENTARY TABLE

Supplementary Table 1: Comparison of Patients' Demographics, Characteristics of Primary Cancer and Lung Metastases and Treatments Between Rectal Cancer and Colon Cancer Patients

See Supplementary File 1
