# Supplementary material for: Post-pulmonary metastasectomy prognosis after curative resection for colorectal cancer
Source: Oncotarget. 2017 Mar 28;8(22):36566–77. doi: 10.18632/oncotarget.16616 (PMC5482677; doi:10.18632/oncotarget.16616)
Supplement: Supplementary file 2 [file oncotarget-08-36566-s002.docx]

Suppl Table 1. Comparison of Patients’ Demographics, Characteristics of Primary Cancer and Lung Metastases and Treatments Between Rectal Cancer and Colon Cancer Patients

A. Patient Demographics and Characteristics of Primary cancer

|  | Colon (N=38) | Rectum (N=91) | p |
| --- | --- | --- | --- |
| **Mean age at metastasectomy, years** | 58 [33-74] | 55 [34-76] | 0.19 |
| **Sex**  Male  Female | 25 (65.8)  13 (34.2) | 52 (57.1)  39 (42.9) | 0.36 |
| **DFI to lung metastasis**  <12 months  ≥12 months | 3 (7.9)  35 (92.1) | 11 (12.1)  80 (87.9) | 0.85 |
| **Primary stage**  0 (NRT)  I  II  III | 3 (7.9)  20 (52.6)  15 (39.5) | 4 (4.4)  16 (17.6)  25 (27.5)  46 (50.4) | 0.59 |
| **Adjuvant chemotherapy for**  **primary cancer**  None  5-FU based  Capecitabine  Others  Not known | 5 (13.2)  9 (23.7)  19 (50.0)  4 (10.5)  1 (2.6) | 2 (2.2)  47 (51.6)  33 (36.3)  7 (7.7)  2 (2.2) | 0.53 |

Data are presented as n [range] or n (percent).

DFI = disease-free interval; NRT = no residual tumor; FU = fluorouracil.

B. Lung Metastases Characteristics and Treatments

|  | Colon (N=38) | Rectum (N=91) | p |
| --- | --- | --- | --- |
| **Location of lung metastasis**  Unilobar  Bilobar | 37 (97.4)  1 (2.6) | 83 (91.2)  8 (8.8) | 0.12 |
| **Number of lung metastasis**  Single  Multiple | 28 (73.7)  10 (26.3) | 73 (80.2)  18 (19.8) | 0.77 |
| **Size of lung metastasis**  <1 cm  ≥1 cm | 10 (26.3)  28 (73.7) | 33 (36.3)  58 (63.7) | 0.14 |
| **Type of resection**  Wedge resection  Segmentectomy  Lobectomy  Combined | 26 (68.4)  6 (15.8)  3 (7.9)  3 (7.9) | 79 (86.8)  5 (5.5)  6 (6.6)  1 (1.1) | 0.08 |
| **CEA level before lung resection**  Normal  Elevated (> 5ng/ml) | 33 (86.8)  5 (13.2) | 82 (90.1)  9 (9.9) | 0.59 |
| **Neoadjuvant chemotherapy for**  **lung metastasis**  Yes  No | 3 (7.9)  35 (92.1) | 6 (6.6)  85 (93.4) | 0.79 |
| **Adjuvant chemotherapy after lung resection**  Yes  No | 29 (76.3)  9 (23.7) | 70 (76.9)  21 (23.1) | 0.85 |

Data are presented as n [range] or n (percent).

CEA = carcinoembryonic antigen.
